# Reconstruction and validation of entire virus model with complete genome from mixed resolution cryo-EM density

**DOI:** 10.1039/d2fd00053a

**Published:** 2022-04-12

**Authors:** Vladimir S. Farafonov, Michael Stich, Dmitry Nerukh

**Affiliations:** a Department of Physical Chemistry, Kharkiv National University Ukraine; b Area of Applied Mathematics, Universidad Rey Juan Carlos Madrid Spain; c Department of Mathematics, Aston University Birmingham UK D.Nerukh@aston.ac.uk

## Abstract

It is very difficult to reconstruct computationally a large biomolecular complex in its biological entirety from experimental data. The resulting atomistic model should not contain gaps structurally and it should yield stable dynamics. We, for the first time, reconstruct from the published incomplete cryo-EM density a complete MS2 virus at atomistic resolution, that is, the capsid with the genome, and validate the result by all-atom molecular dynamics with explicit water. The available experimental data includes a high resolution protein capsid and an inhomogeneously resolved genome map. For the genomic RNA, apart from 16 hairpins with atomistic resolution, the strands near the capsid’s inner surface were resolved up to the nucleic backbone level, and the innermost density was completely unresolved. As a result, only 242 nucleotides (out of 3569) were positioned, while only a fragmented backbone was outlined for the rest of the genome, making a detailed model reconstruction necessary. For model reconstruction, in addition to the available atomistic structure information, we extensively used the predicted secondary structure of the genome (base pairing). The technique was based on semi-automatic building of relatively large strands of RNA with subsequent manual positioning over the traced backbone. The entire virus structure (capsid + genome) was validated by a molecular dynamics run in physiological solution with ions at standard conditions confirming the stability of the model.

## Abstract 2

We, for the first time, reconstruct from the published incomplete cryo-EM density a complete MS2 virus at atomistic resolution, that is, the capsid with the genome, and validate the result by all-atom molecular dynamics with explicit water.

## Graphical abstract

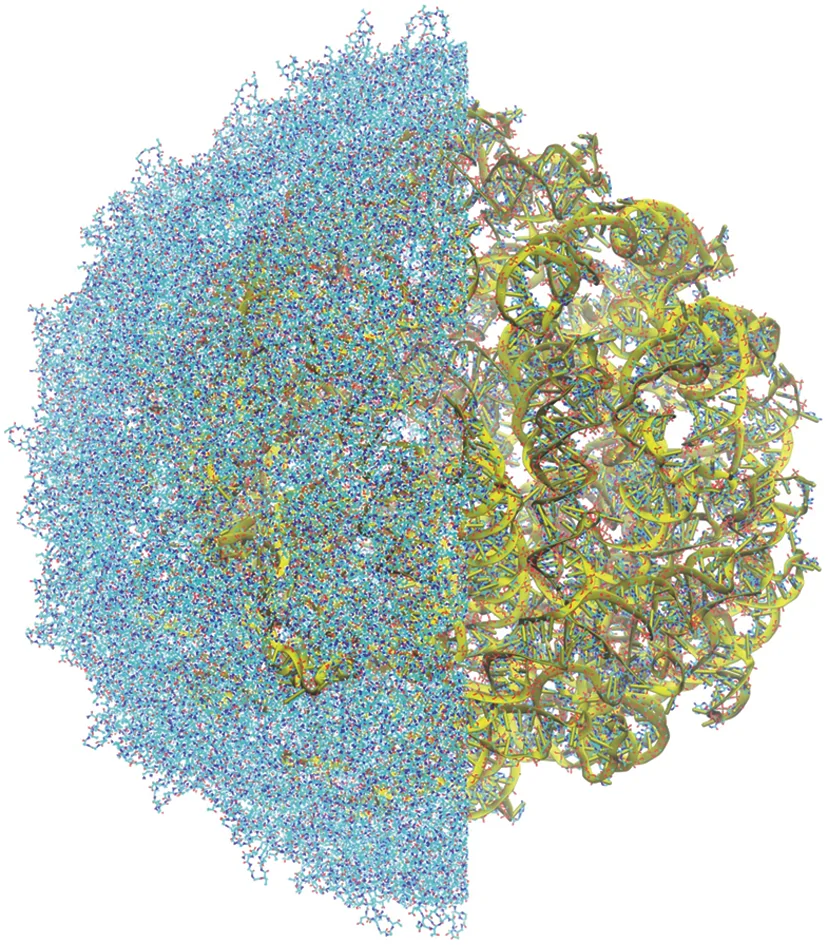

## Introduction

State of the art experimental techniques allow the measurement of the cryo-EM maps of biomolecular systems at a resolution sufficient for reconstructing the atomistic structure of the molecules. However, such reconstruction (“fitting”) is a non-trivial procedure, especially for maps with non-uniform resolution (see ref. 1 and 2 for recent examples). The problem is particularly challenging for large biomolecular systems, such as entire viruses or cellular organelles, because typical algorithms are usually incapable of fitting the whole structure at once and non-trivial approaches are needed to fit parts of the system and build a coherent complete structure.

The structures of virus particles have been measured for a number of different viruses recently.^3–10^ In the majority of the published data, however, only the protein capsid of the virus is measured. This is because the resolution of the measured cryo-EM map for the interior of the virus is typically insufficient for fitting the atomistic structure. One of the most studied viruses is the bacteriophage MS2, for which an incomplete cryo-EM density has been reported,^6^ although this included some structural information about its genome. Despite an existing attempt,^11^ where the genome was partially reconstructed, a *complete* native genome model has never been published nor validated.

We here suggest an approach to reconstruct the atomistic structure of MS2 in its entirety, including the native genome. We use as much information as possible from the measured cryo-EM structure, the chemical (primary) structure of the genome, and the secondary (base pairing) structure of the genomic RNA. When the model is built, we validate it by performing a molecular dynamics simulation of the virus in solution at physiological conditions.

## Methods

### Input data overview

According to the cryo-EM experiment,^6^ the collected density of the MS2 virion has an inhomogeneous resolution. For the capsid, an almost complete atomistic reconstruction of the coat proteins and one maturation protein (MP) was achieved. The first missing part is in one coat protein, lacking 10 amino acid residues located on the outer surface, which we reconstructed previously.^12^ Secondly, four outermost intervals of the maturation protein are missing, 63 residues in total, which are most probably too flexible to be measured with atomistic resolution.

The genome map has three levels of detail. Firstly, there are 16 approximately 15-nucleotides long segments resolved with atomistic resolution. These are stem-loops in contact with the internal surface of the capsid or the MP. Secondly, the density near the inner surface is resolved up to the level of the RNA backbone, providing the trace of its tertiary structure. Finally, the innermost density is not resolved at all. Summarising, the accurate position of 242 nucleotides and the approximate location of more than half of the genome backbone are known.

The secondary structure of the genome (base pairing) is known precisely for the 16 completely resolved stem-loops. For the rest, it was predicted computationally.

### Genome reconstruction

The scale of the problem and the resolution of the density rendered the commonly used method of molecular dynamics flexible fitting inappropriate for the task. Instead, we used the resolved backbone together with the secondary structure prediction (SSP) as much as possible. Overall, our technique is based on the semi-automatic building of relatively large pieces of RNA and further positioning them manually over the backbone trace provided by the cryo-EM experiment. The detailed reconstruction procedure is presented in the Appendix, and here we describe the general outline of our approach.

The first stage consists of preparing a piece-wise approximation of the genome. It is done according to the following algorithm (starting from the first nucleotide).

(1) Pick a primary structure segment starting from the current nucleotide. The length of the segment is determined from the SSP to include one complete single or double helix. The double helix may be a stem-loop, but this is not necessary.

(2) Generate a perfect all-atom A-form double or single helix for this nucleotide sequence using one of the modelling approaches. We used the Make-NA web server for this purpose.^13^

(3) If а double helix was generated, correct the placement of individual nucleotides to make hairpins and bulges, if they are present in the SSP. We used residue movement/rotation capabilities of the VMD program for this.^14^

(4) Place the prepared structure in such a way that the following requirements are met: (i) its backbone matches the experimental trace; (ii) its first nucleotide is near the last nucleotide of the previous piece; (iii) it does not clash with the capsid and the pieces placed before. If needed, slightly shift the surrounding pieces. The two strands of the double helix may be adjusted independently, provided that their complementarity is satisfied. A common difficulty is that long stem-loops are usually curved, while the generated ones are straight. In these cases, the beginning of the piece should mainly be matched, while fitting the ends may be left for the later stages. If there is no experimental backbone available for the piece, then the only requirements to follow are (ii) and (iii). We did it manually by eye using VMD.

(5) If at step (4) all the requirements are fulfilled, then return to step (1) with the next piece.

(6) Otherwise, if the match between the piece and the trace is questionable (*e.g.* the piece is a stem-loop and has more or less turns than the corresponding interval of the backbone trace), then a detailed analysis must be done. There could be two types of problems revealed by the analysis. On one hand, the trace may be incomplete, thus, it becomes justified to extend the piece beyond it and go to step (5). On the other hand, the SSP may be inaccurate, therefore, other variants of the secondary structure of this segment must be considered, and the algorithm returns to step (1) with the same starting nucleotide but using another SSP. We employed the MC-Fold|MC-Sym pipeline for folding the RNA sequences.^15^

As a result of this algorithm, a set of RNA pieces is produced and placed, which has several crucial features: it

(i) covers the complete sequence,

(ii) closely follows the resolved backbone,

(iii) has a reasonable secondary structure,

(iv) possibly has short gaps between the ends of the neighbouring pieces,

(v) has no or only very few steric clashes.

In our case, there were 143 pieces in total. An example of a perfect RNA piece with consequent correction is shown in Fig. 1, and the placed pieces covering the segment 614–887 nucleotides is shown in Fig. 2. Essentially, the obtained structure is a rough model of the complete genome that has some distortions:

(i) the bond lengths and angles between the nucleotides belonging to neighbouring pieces differ from their equilibrium values because they were aligned approximately and

(ii) some steric clashes between pieces are still present.

**Fig. 1:**
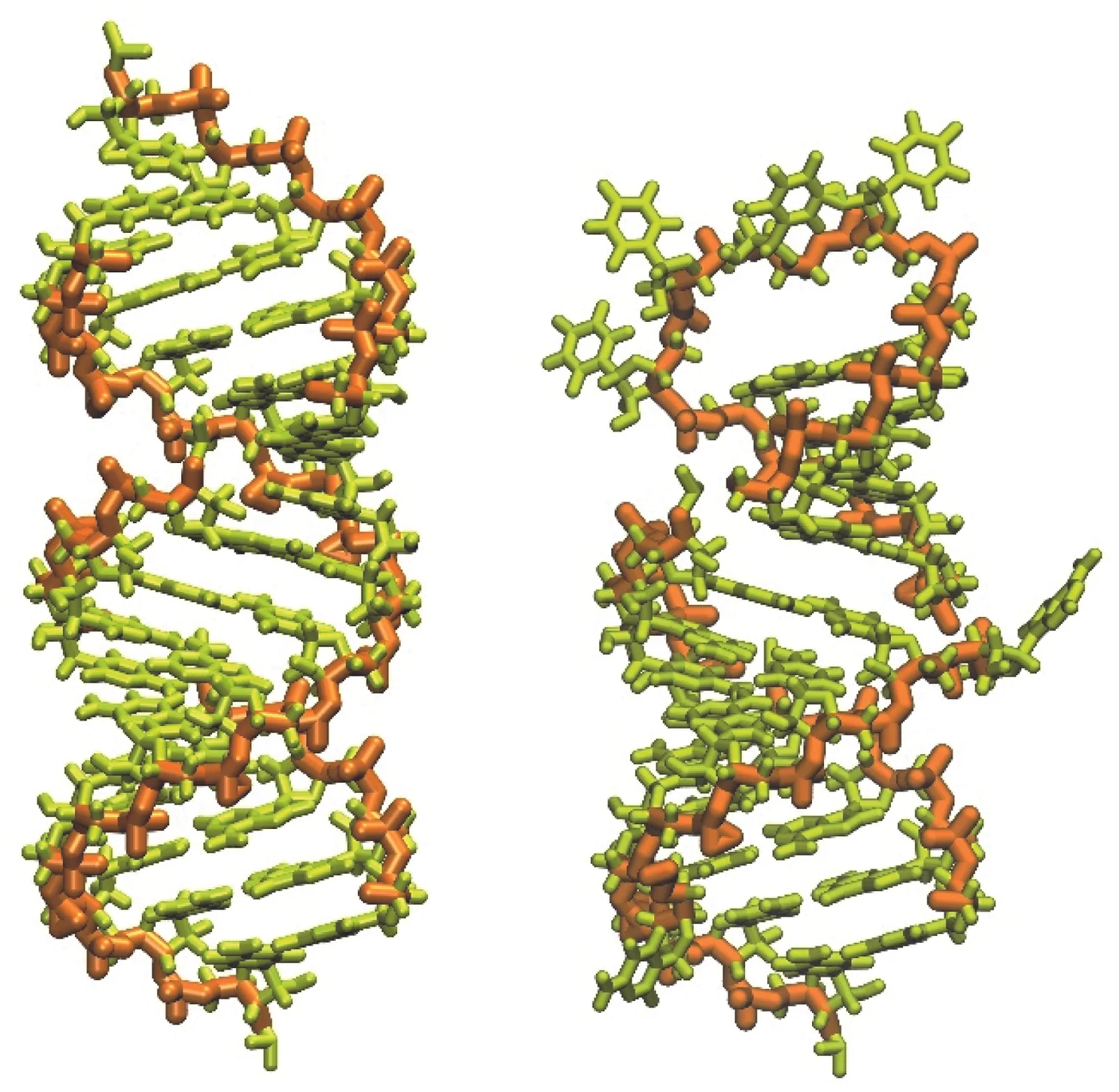
RNA piece 2057–2085, its generated double-strand all-atom structure (left) and corrected hairpin all-atom structure (right). The nucleic backbone is highlighted in orange.

**Fig. 2:**
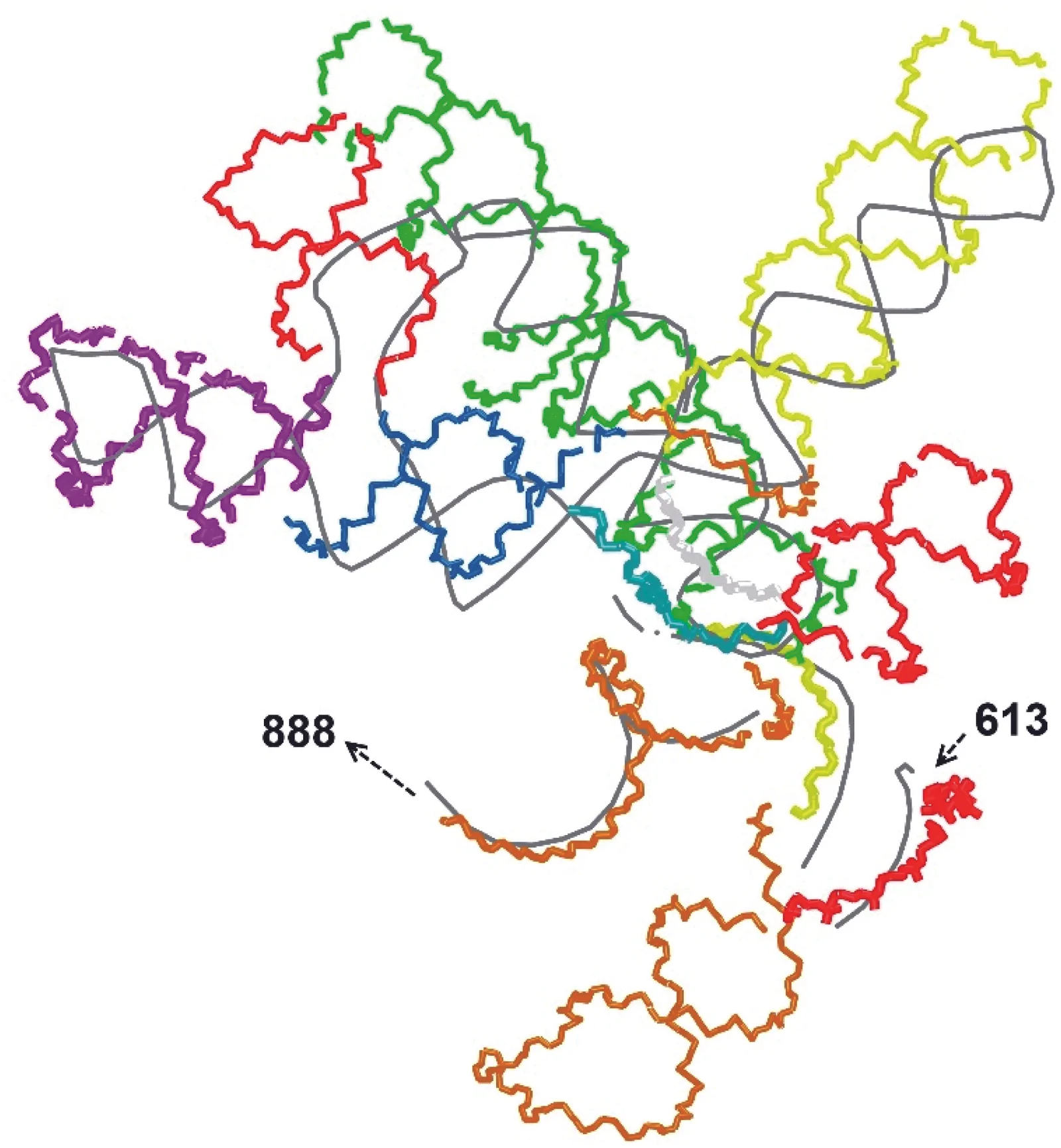
A set of 14 RNA pieces covering the segment 614–887 manually aligned over the experimental trace (grey). The pieces are coloured in the following sequence: red, orange, yellow, green, light blue, blue, violet, red, blue, orange, yellow, light gray, red, orange. Only the backbones are shown.

At the second stage, the piece-wise model is made continuous.

(1) The individual pieces are concatenated to a single molecule.

(2) The structure is relaxed to remove the distortions. In our case, the molecule appeared to not be suitable for energy minimisation at all-atom resolution because the defects were too large for the common algorithms, like the steepest descent method. A way to circumvent this difficulty is to turn to the coarse-grained level for relaxing the structure. After the relaxation is done, the coarse-grained model is backmapped to the atomistic one. The CafeMol program was used to relax and backmap the coarse-grained genome model,^16^ see the Appendix for details.

(3) The produced all-atom structure is free from severe defects and is thus acceptable for routine energy minimisation. This operation was done using the GROMACS package.^17^

(4) The last deficiency of the model is that it cannot be fitted into the capsid due to the protruding long straight stem-loops, which *in vivo* are aligned along the capsid inner wall. This problem was fixed with a pulling run in GROMACS, see the Appendix for details.

Using this algorithm, a complete, accurate, and operable all-atom model of MS2 genome was created, ready for molecular dynamics simulations.

## Results and discussion

The complete prepared atomistic model of MS2 genome is presented in Fig. 3. Fig. 5–12 depict the separate segments of the model together with their secondary structure; the experimental traced backbone is shown for comparison. We applied the same segmentation as in the experimental paper.^2^ The model reproduces all structural elements of the latter, that is the number and length of the stem-loops and their positioning relative to each other. Furthermore, it contains the completely non-resolved segments, for which a sound folding is imposed, following the methods described above and in the Appendix. The deviations from the trace are mostly caused by the flexibility of long stem-loops. The secondary structure to a large extent matches the prediction of the experimental paper, namely, only 24% nucleotides in our model have different pairing.

**Fig. 3:**
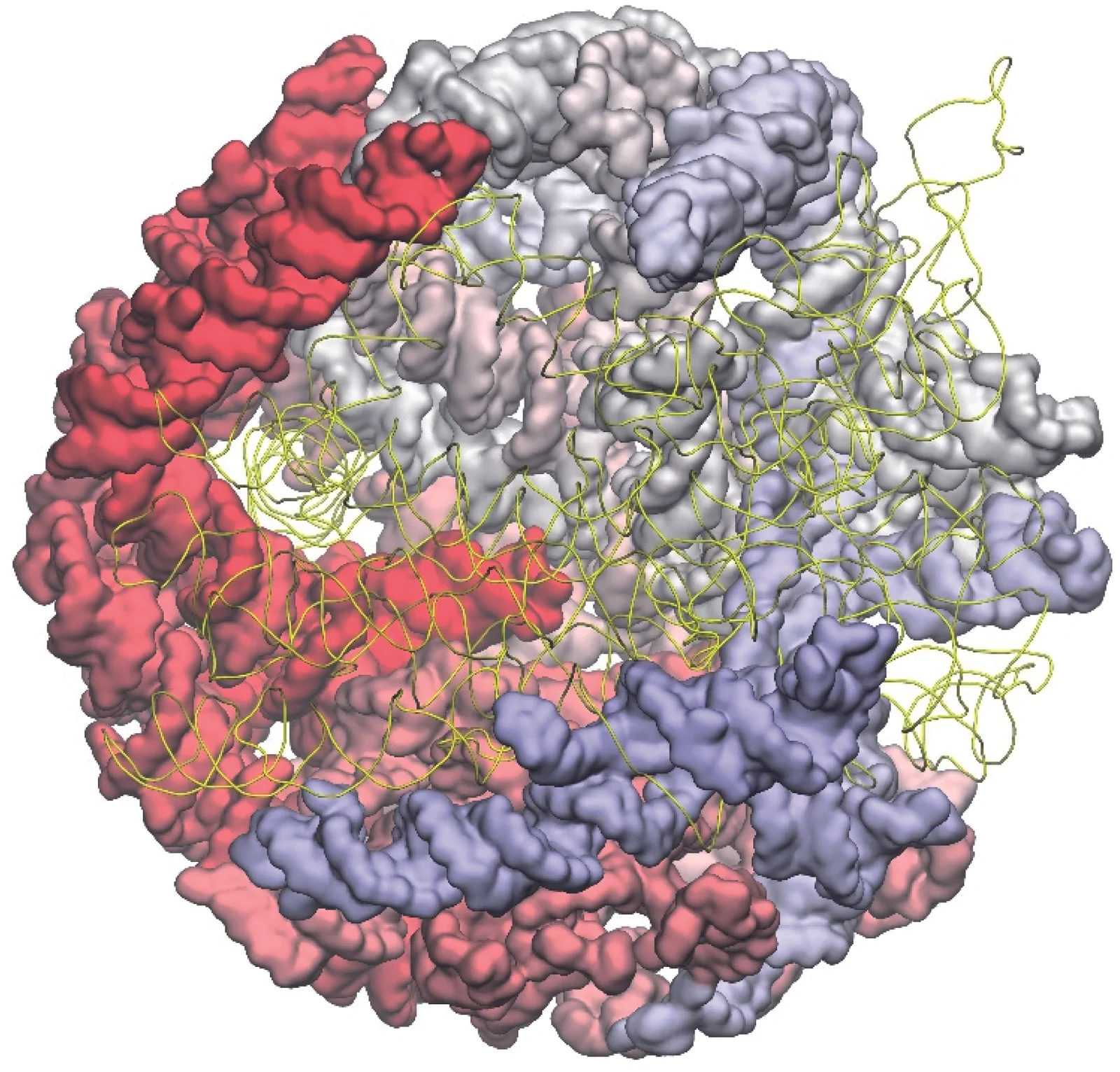
The all-atom model of the MS2 genome as reconstructed from cryo-EM data. The residues 1–2500 are represented as surfaces coloured by the residue number red to white to blue; the residues 2501–3569 are shown as yellow strands to reveal the interior of the structure.

The operability and stability of the prepared model was tested in the most relevant conditions. It was placed within the atomistic assembled MS2 capsid solvated in physiological saline. The complete virus particle was assembled at several stages, largely following the algorithm used in our paper^4^ on reconstructing the MS2 capsid, Fig. 4. Then, an MD run was carried out for 50 ns at 298 K, mimicking laboratory conditions. The 3D periodic boundary conditions were imposed, the time step was 2 fs, and all covalent bonds were constrained by the LINCS algorithm. Electrostatic interactions were computed with the PME method, while the van der Waals interactions were cut off at 1 nm.

**Fig. 4:**
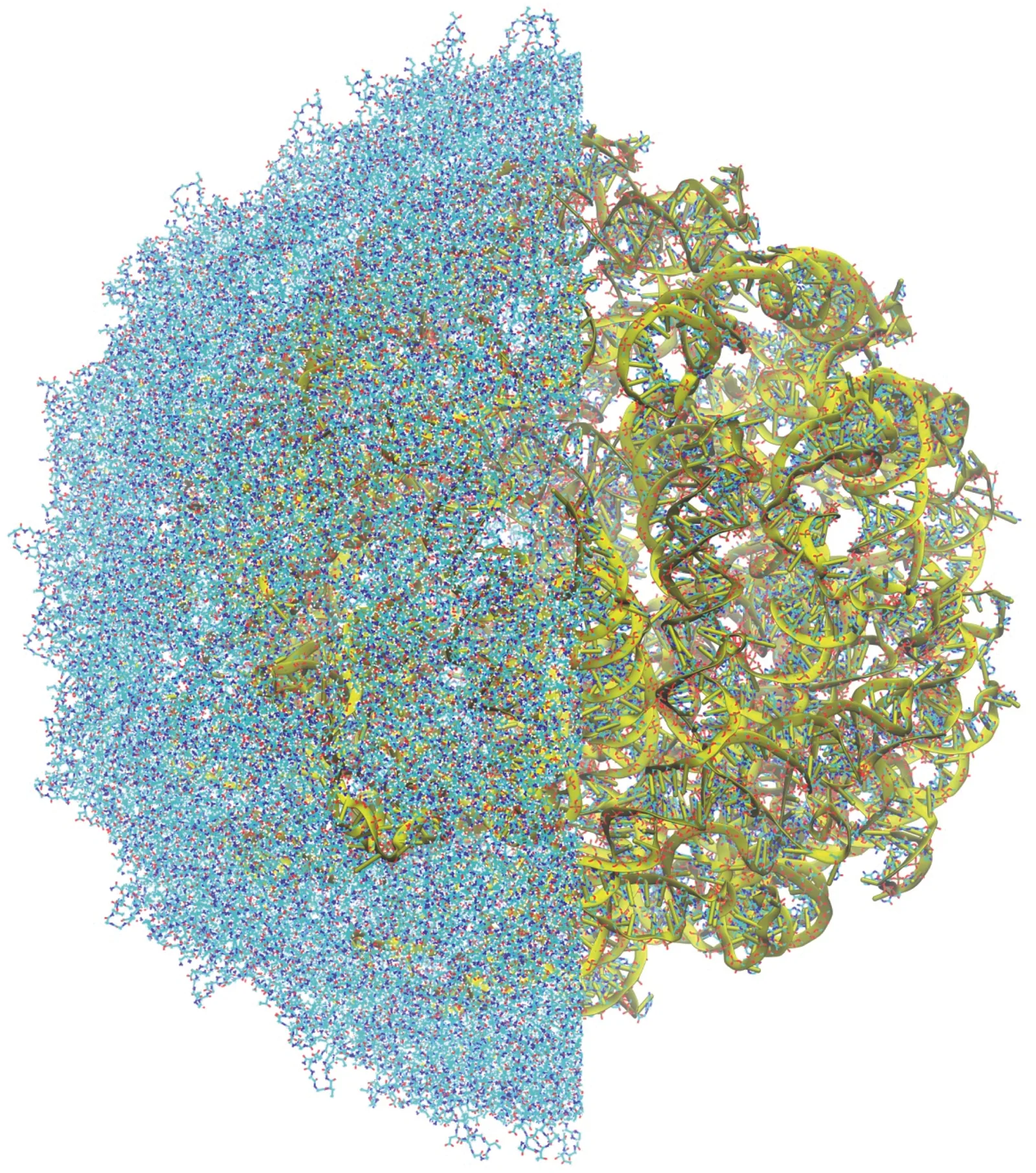
Atomistic structure of the complete MS2 bacteriophage consisting of the protein capsid and genomic RNA. Half of the capsid is cut to reveal the genome; the backbone of the genome is shown in yellow.

During the simulation, the model showed limited deviation from its initial configuration, which indicates the absence of significant stresses and unnatural structural motifs. Quantitatively, the root-mean-square displacement after 50 ns was equal to 0.8 nm, of which 0.3 nm occurred during the first nanosecond.

## Conclusions

In conclusion, we successfully developed an approach for fitting an atomistic structure to incomplete and highly heterogeneous in resolution experimental data of an entire MS2 virus particle, including its native genome. The resulting structure is MD ready and we have validated it by performing a standard MD equilibration followed by an MD run for 50 nanoseconds. To the best of our knowledge, this is the first complete reconstruction with native genome and all-atom MD simulation of an MS2 bacteriophage or any other virus. We expect that our algorithms can be applied to other cryo-EM reconstruction problems with incomplete data.

## Appendices

### Assembling the piece-wise model

Perfect all-atom A-form structures of RNA pieces were generated using the Make-NA web server.^5^ The obtained pdb files were processed as follows.

(1) The 5′ H atom of the first nucleotide was removed.

(2) The 3′ H atom of the last nucleotide was replaced with a PO_3_ group.

(3) The placeholders (labelled as “N” residues) were deleted, leaving gaps in the structure.

(4) Nucleotides on the free ends were manually positioned to form a stem-loop if needed.

(5) The structure was manipulated (bent) to remove gaps if present.

Steps (1) and (2) were needed to facilitate the subsequent joining of the parts. For visualisation and manipulating the RNA pieces, the VMD software was used.^6^ For manual operations, a perfect accuracy was not needed because the structure was relaxed at later stages.

### Assembling the continuous model

The double helices, which do not form stem-loops, were split into two strands. Then, the individual pieces were joined to a single pdb file in the correct order.

### Coarse-grained relaxation

The CafeMol 3.1 program^8^ was used for all coarse-grained (CG) operations. In its representation, a nucleotide contains three interaction sites: one corresponding to the sugar moiety, the second one corresponding to the base, and third one corresponding to the phosphate linkage. Therefore, the CG model was prepared by generating the three interaction sites for each nucleotide, with one placed in the centre of the mass of the sugar ring, the second in the centre of mass of the base, and the third in the position of the P atom.

The second step was the preparation of the native structure information, that is the list of all intramolecular interactions between the CG sites of the nucleotides. Importantly, the CafeMol representation contains both bonded (bonds, angles, dihedral angles) and non-bonded (stacking, base-pairing) interactions. As a result, not only the primary structure will be recovered during the simulation (meaning the valence distances and angles will be brought to their equilibrium values), but also the specified secondary structure will be imposed (the nucleotides indicated as paired will be brought together, while non-paired will be repulsed from each other regardless of their potential ability to pair). This behaviour is particularly well suited for the task of relaxing the rough manually prepared structure because the desired secondary structure is known. The information was prepared using our own scripts.

The simulation was carried out at a constant temperature of 30 K with the a time step of 0.1 CafeMol units for 50 000 steps in physiological solution imitated by a continuum. The resulting configuration had the root-mean-square deviation of 0.6 nm with respect to the initial structure.

Finally, the CafeMol backmapping tool was used to restore the all-atom model. Because it is suited for DNA, not RNA, in the relaxed CG model the nucleotides were renamed to their deoxy-analogues, and after backmapping the all-atom DNA model was converted to RNA by replacing the corresponding H atoms with OH groups. It was found to be necessary to split the CG model to two halves and backmap each of them separately, with subsequent joining, to meet the software limitations.

### All-atom refinement

The stem-loops used for building the starting piece-wise model were straight, and this shape was kept in the relaxed all-atom model. This prevents it from fitting into the capsid because some stem-loops are very long and thus would simply protrude through the capsid. In reality, such stem-loops are curved to match the capsid inner wall. To impose the correct shape, an MD run was performed using the GROMACS suite.^9^ The genome was surrounded by the atoms of the inner side of the capsid, which were initially placed at a distance 5 nm farther from the capsid centre than they are located in the experimental structure. The capsid atoms were made non-interacting with each other, but interacting with the genome atoms. In the MD run, they were made to be moving slowly towards their proper positions, thus squeezing the capsid and making the genome fit into the capsid. The run lasted for 200 ps in the absence of solvent and periodic boundary conditions. The movement rate of the capsid atoms was equal to 25 nm ns^−1^, while the RNA was weakly fixed at its initial position by a constant force of 1 kJ mol^−1^ nm^−1^ to prevent its displacement. Importantly, the segment 3540–3569 in the end of the genome representing a resolved stem-loop penetrated the capsid near the maturation protein. Therefore, it was made non-interacting with the squeezing capsid, because otherwise an irresolvable collision would occur. Instead, after squeezing the capsid, it was individually pulled to the position revealed by the cryo-EM measurement. This run was 200 ps long and pulling was done with a constant force of 50 kJ mol^−1^ nm^−1^. The resulting model is the final result of this reconstruction, and it is the one showed in Fig. 5–12.

**Fig. 5:**
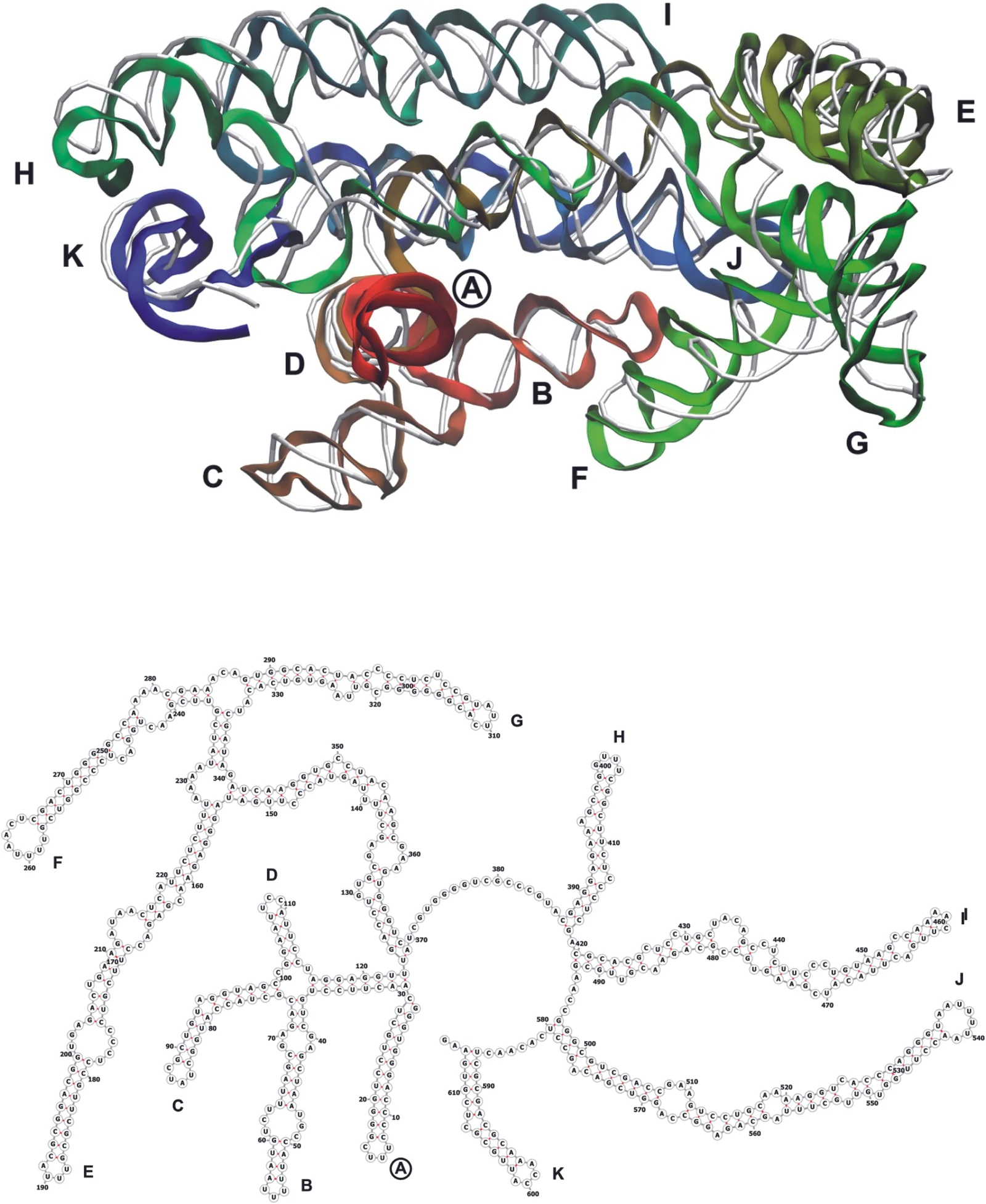
Segment 1–615 of the reconstructed genome model (red to green to blue), placed over the cryo-EM backbone (white), and its secondary structure. The stem-loops with no corresponding cryo-EM backbone are marked with circles.

**Fig. 6:**
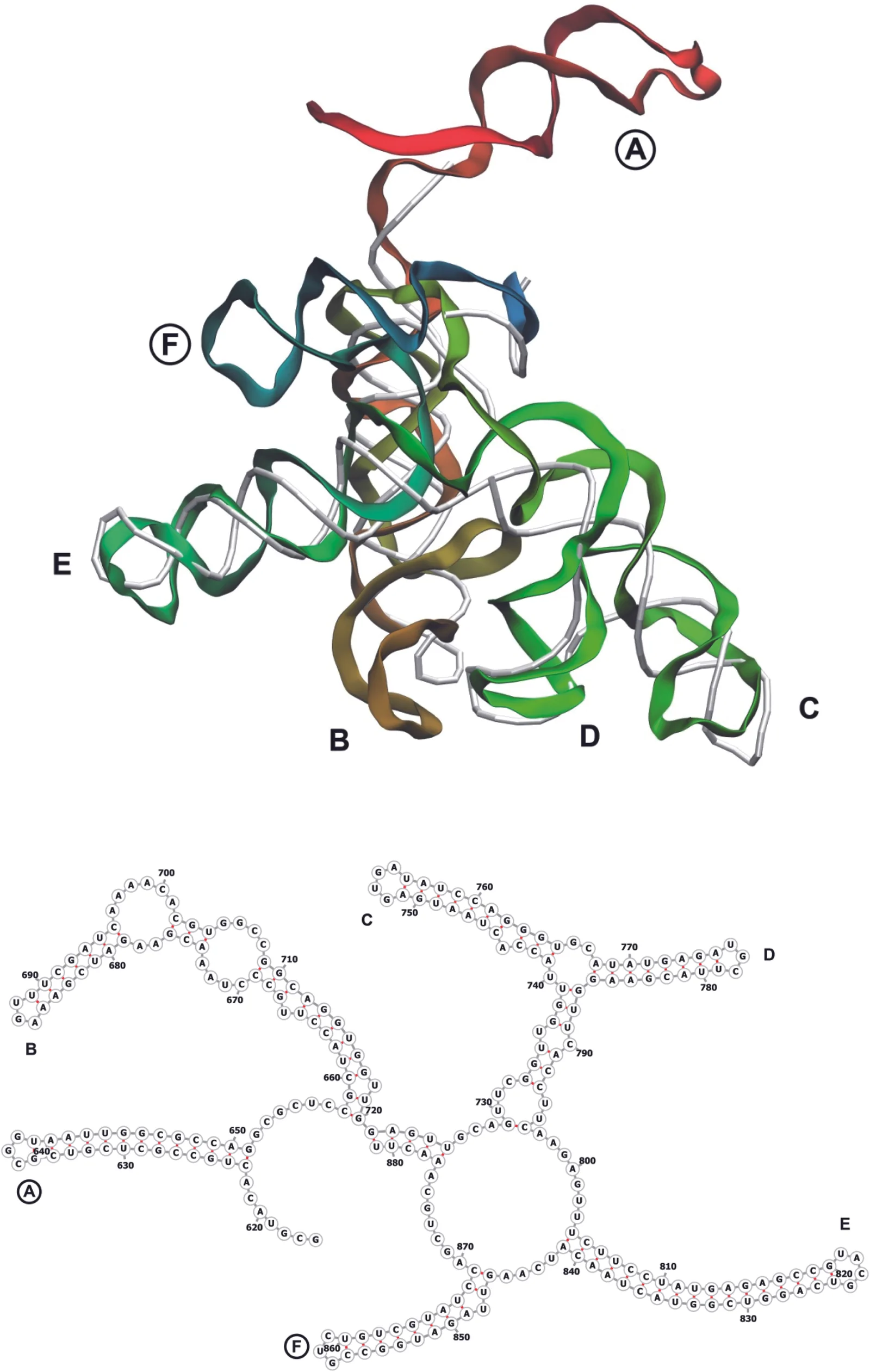
Segment 616–881 of the reconstructed genome model (red to green to blue), placed over the cryo-EM backbone (white), and its secondary structure. The stem-loops with no corresponding cryo-EM backbone are marked with circles.

**Fig. 7:**
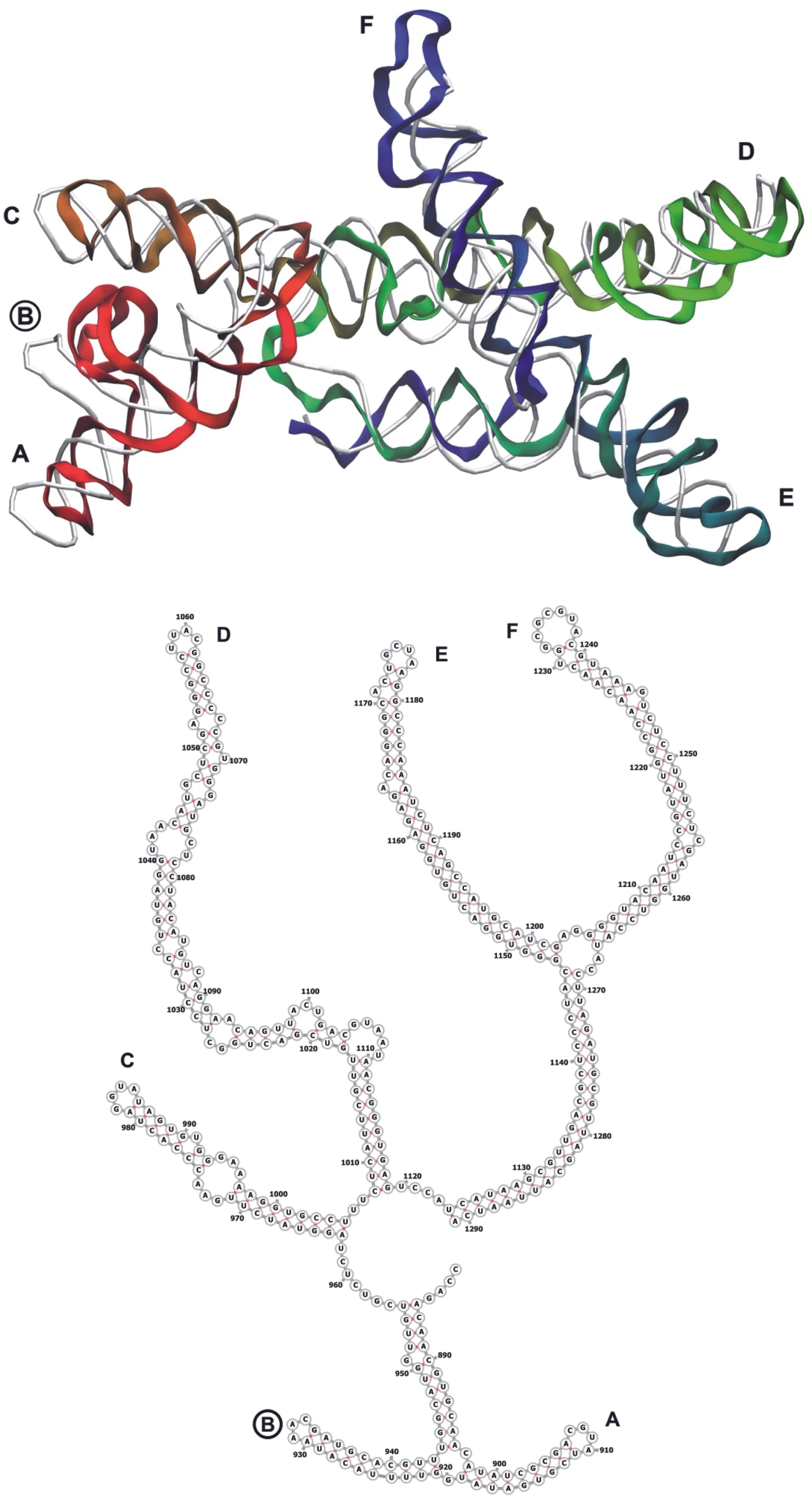
Segment 882–1291 of the reconstructed genome model (red to green to blue), placed over the cryo-EM backbone (white), and its secondary structure. The stem-loops with no corresponding cryo-EM backbone are marked with circles.

**Fig. 8:**
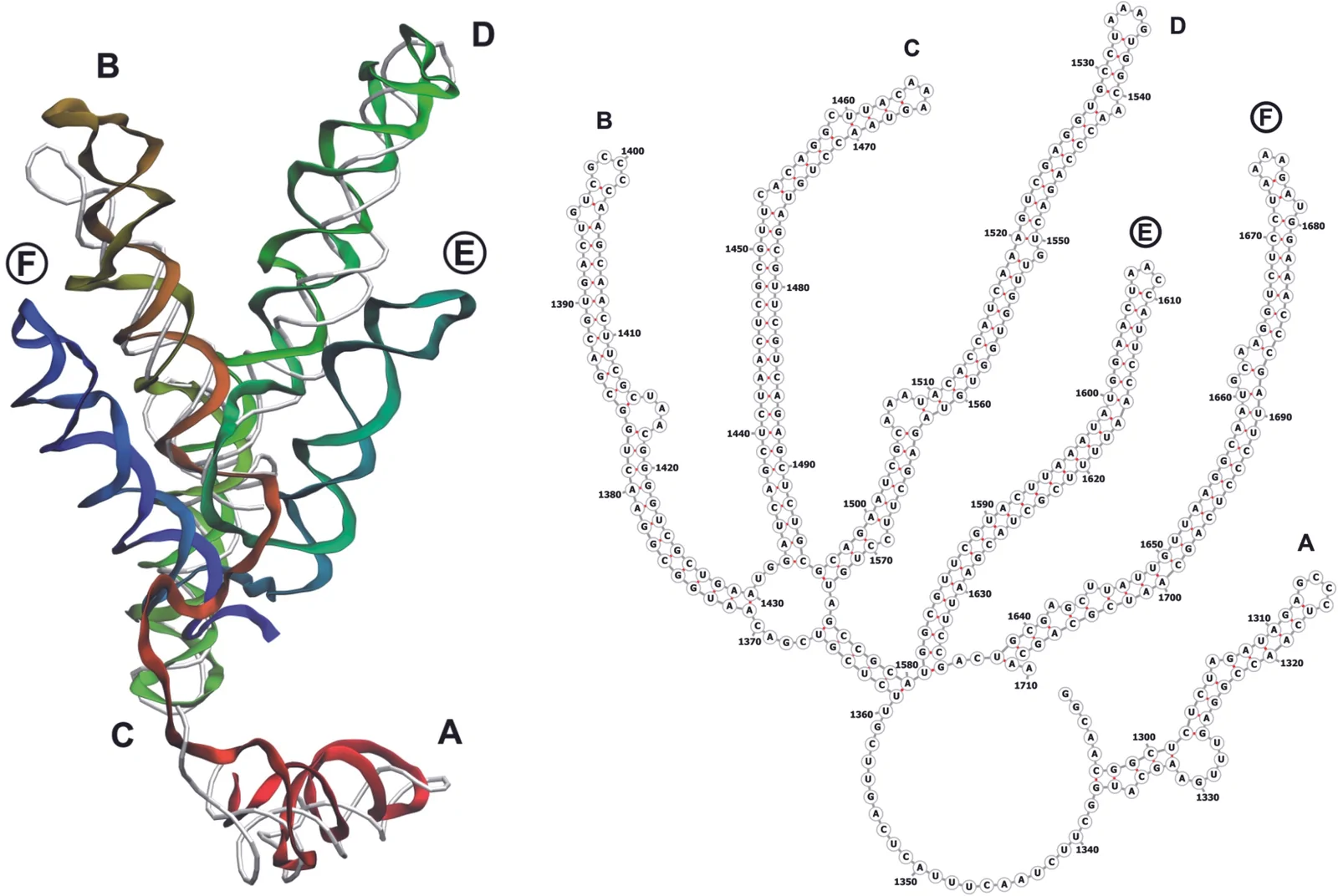
Segment 1292–1710 of the reconstructed genome model (red to green to blue), placed over the cryo-EM backbone (white), and its secondary structure. The stem-loops with no corresponding cryo-EM backbone are marked with circles.

**Fig. 9:**
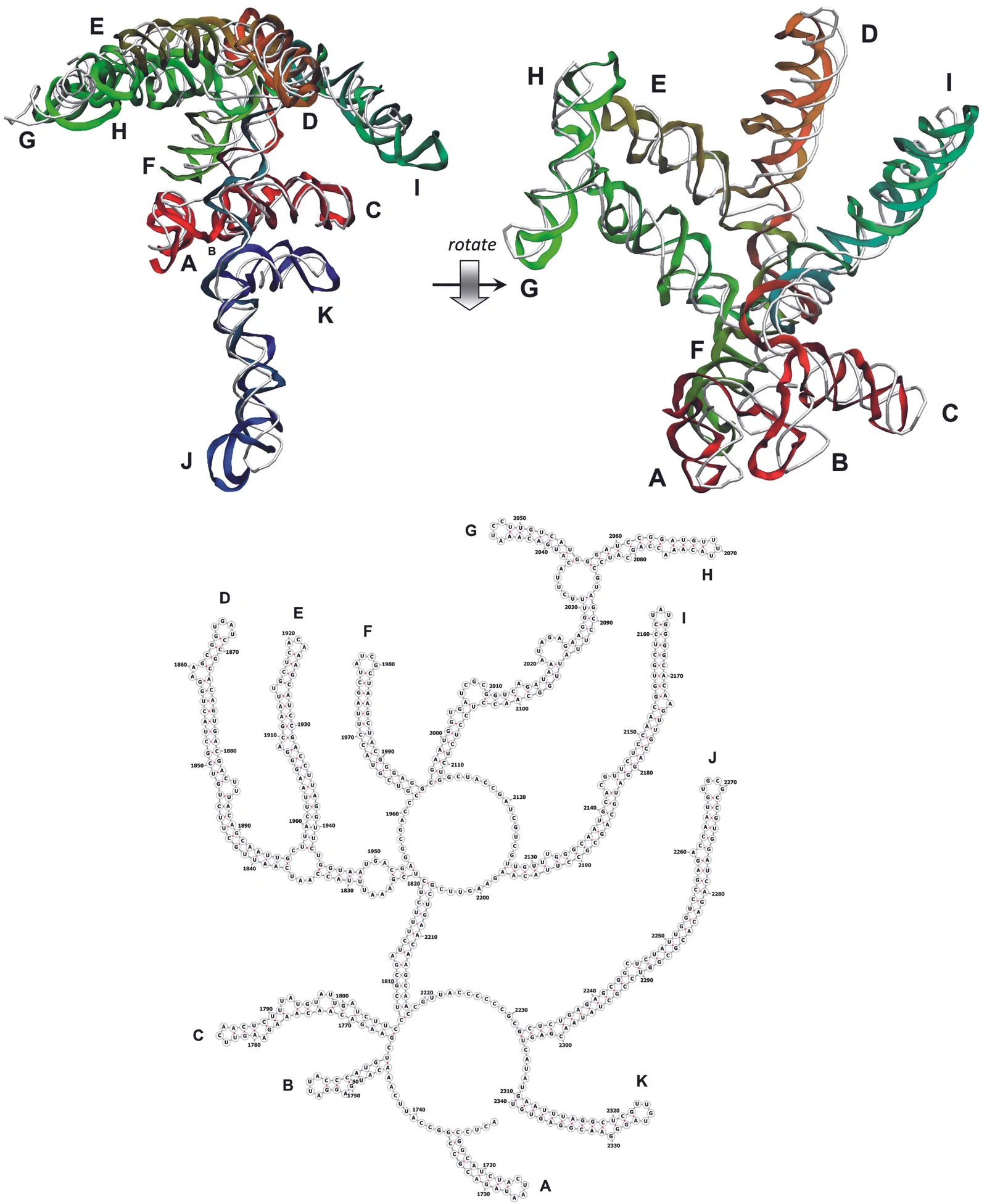
Segment 1711–2340 of the reconstructed genome model (red to green to blue), placed over the cryo-EM backbone (white), and its secondary structure.

**Fig. 10:**
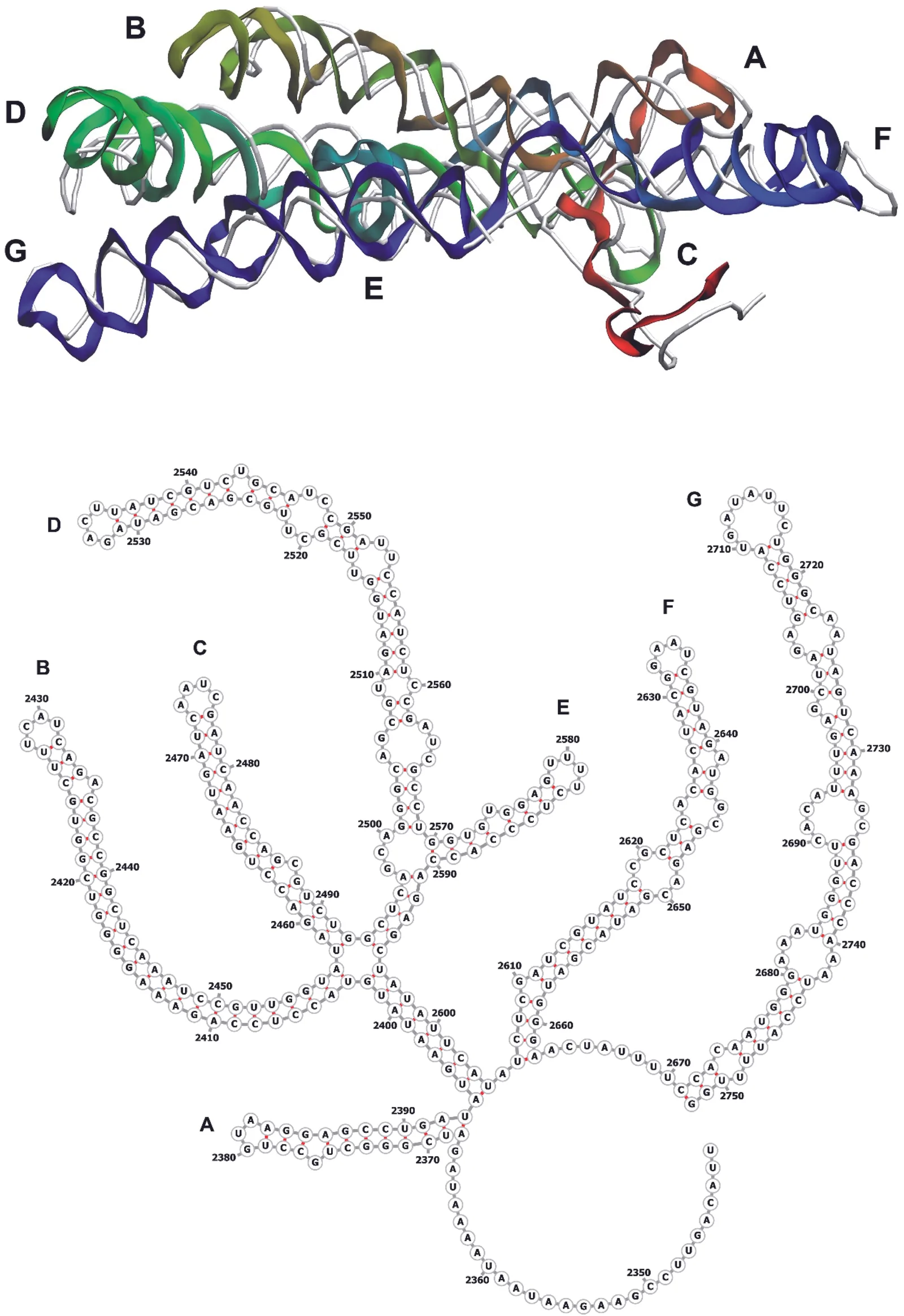
Segment 2341–2752 of the reconstructed genome model (red to green to blue), placed over the cryo-EM backbone (white), and its secondary structure.

**Fig. 11:**
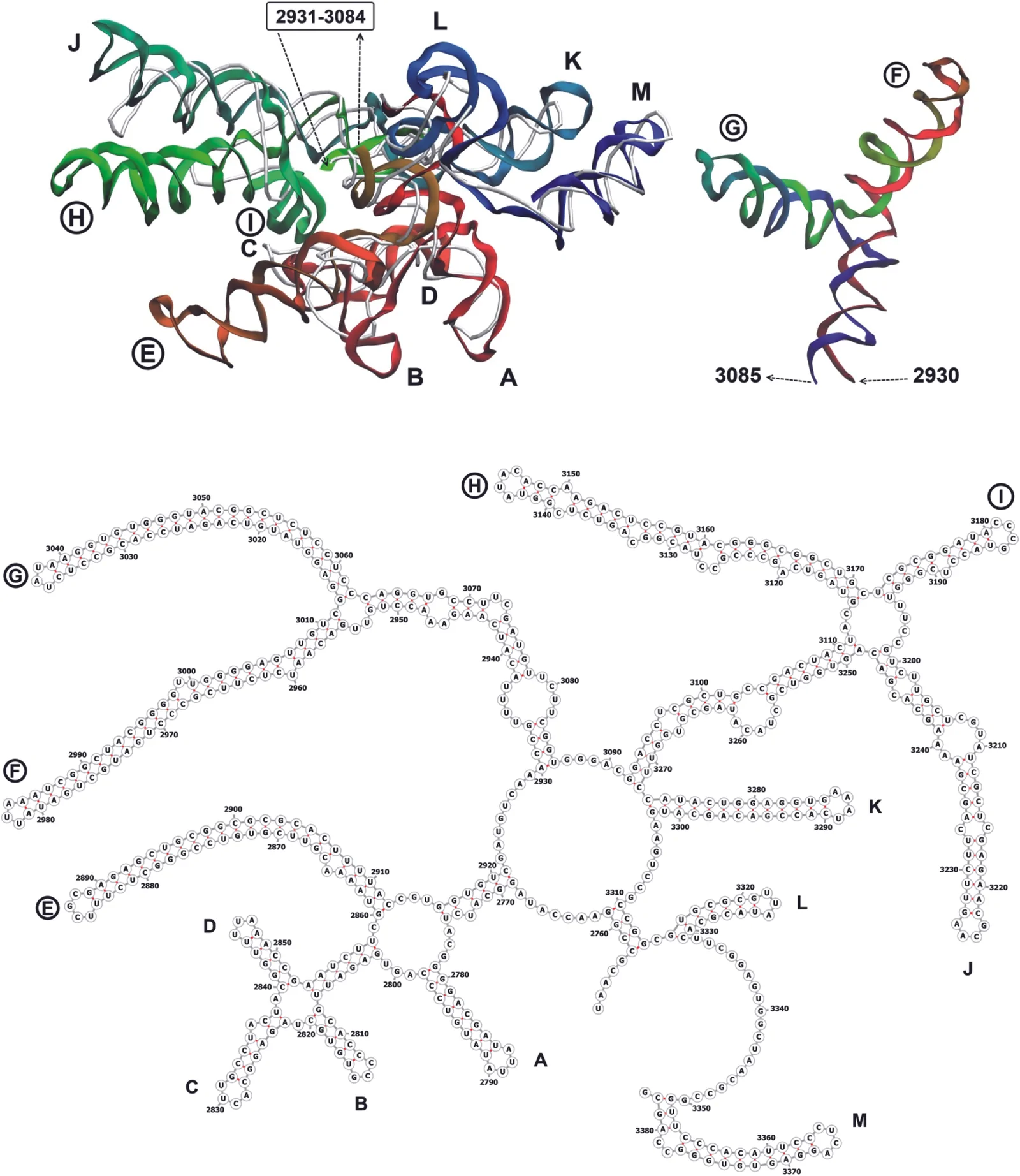
Segment 2753–3383 of the reconstructed genome model (red to green to blue), placed over the cryo-EM backbone (white), and its secondary structure. Interval 2931–3084 is shown separately. The stem-loops with no corresponding cryo-EM backbone are marked with circles.

**Fig. 12:**
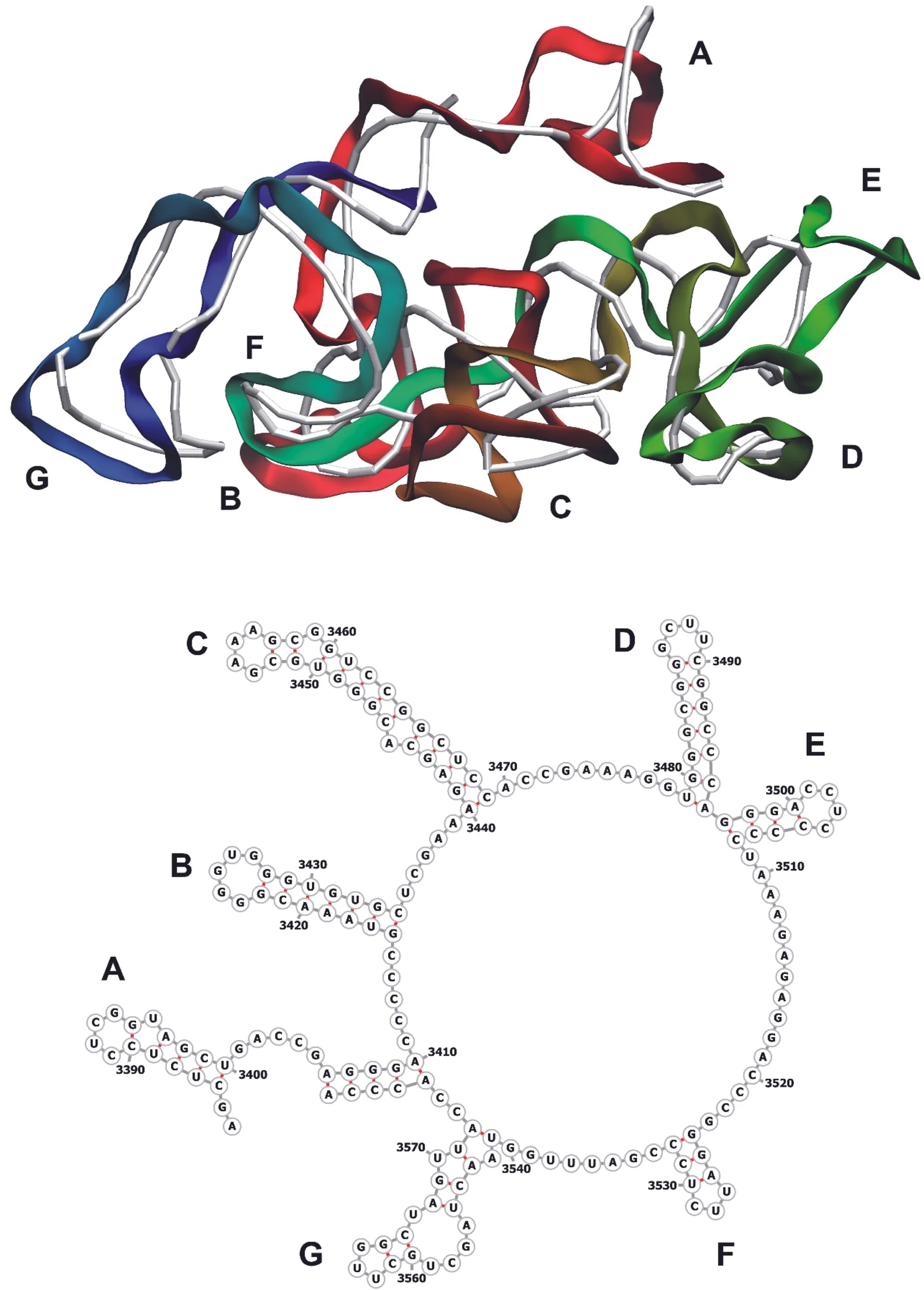
Segment 3384–3569 of the reconstructed genome model (red to green to blue), placed over the cryo-EM backbone (white), and its secondary structure.

## Conflicts of interest

There are no conflicts to declare.
